# Supplementary material for: Improving Stability of Roll-to-Roll (R2R) Gravure-Printed Carbon Nanotube-Based Thin Film Transistors via R2R Plasma-Enhanced Chemical Vapor-Deposited Silicon Nitride
Source: Nanomaterials (Basel). 2023 Jan 30;13(3):559. doi: 10.3390/nano13030559 (PMC9918980; doi:10.3390/nano13030559)
Supplement: Supplementary file 1 [file nanomaterials-13-00559-s001.zip › nanomaterials-2169887-supplementary.pdf]

Supplementary Materials

Improving Stability of Roll-to-Roll (R2R) Gravure-Printed Carbon Nanotube-Based Thin Film Transistors via R2R Plasma-Enhanced Chemical Vapor-Deposited Silicon Nitride

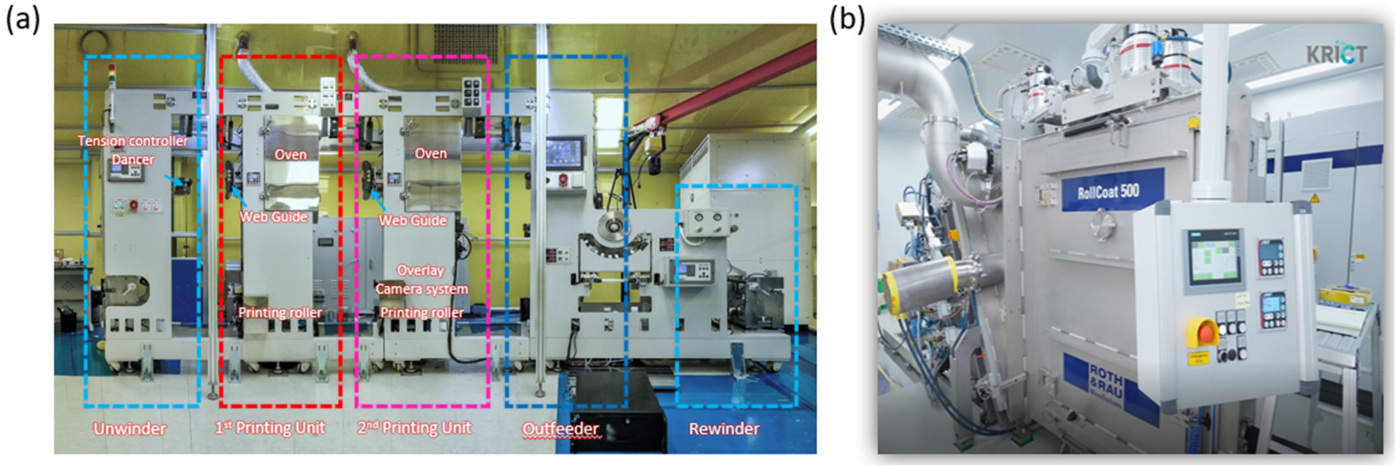

Figure S1. (a) R2R gravure printing system with two printing units. (b) R2R plasma-enhanced chemical vapor deposition system.

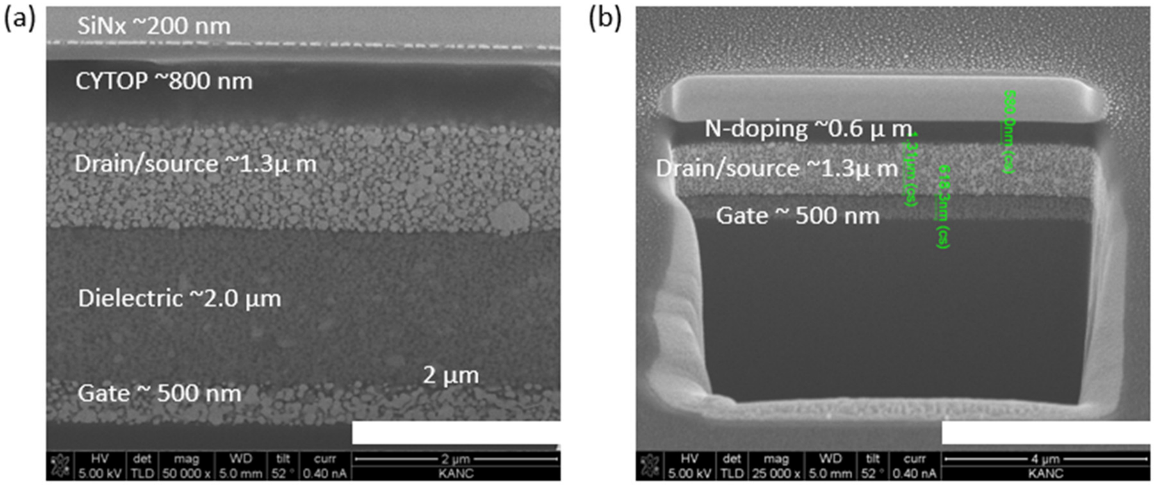

Figure S2. (a) The cross-sectional scanning electron microscope image of R2R printed p-type SWCNT-TFT with SiNx passivation layer. (b) The cross-sectional scanning electron microscope image of R2R printed n-type SWCNT-TFT with an n-doping layer.

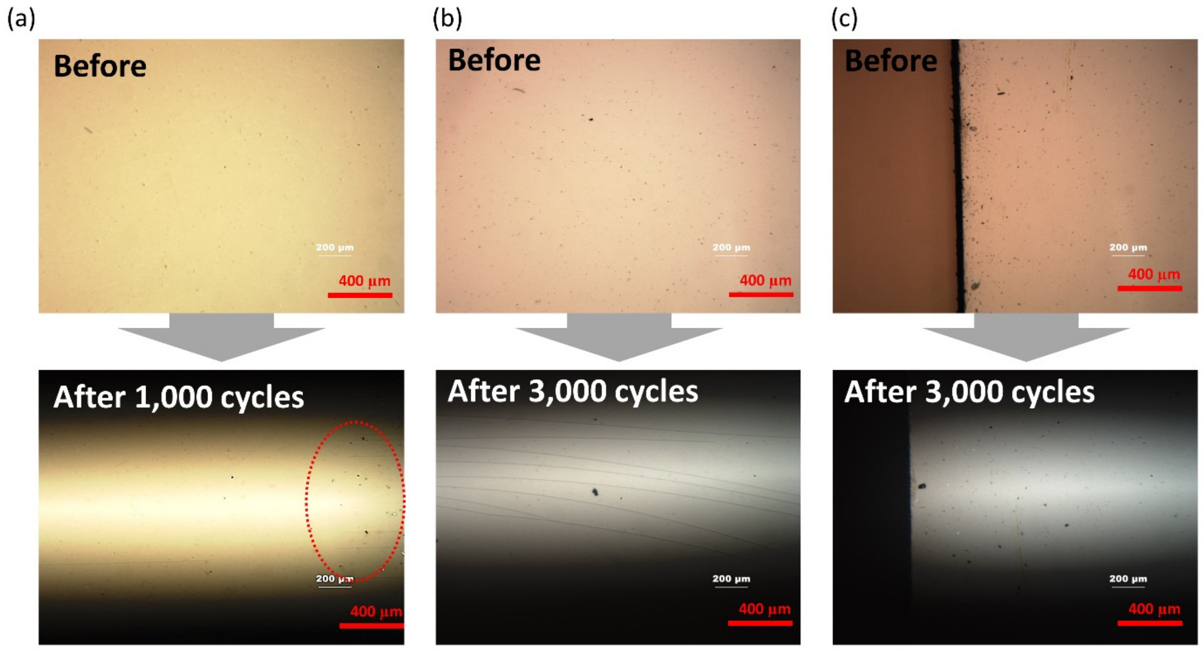

Figure S3. Optical images of before and after bending test for SiNx layer on PET with thickness (a) 400 nm (bending test for 1,000 cycles) , (b) 300 nm (bending test for 3,000 cycles) and, (c) 200 nm (bending test for 3,000 cycles).

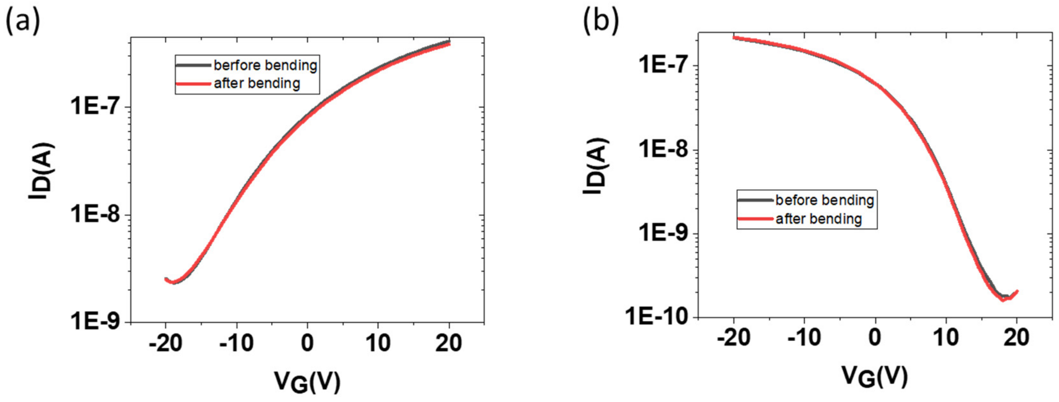

Figure S4. Transfer characteristics of SiNx passivated (a) n-type and, (b) p-type pSWCNT-TFTs before and after bending tests.

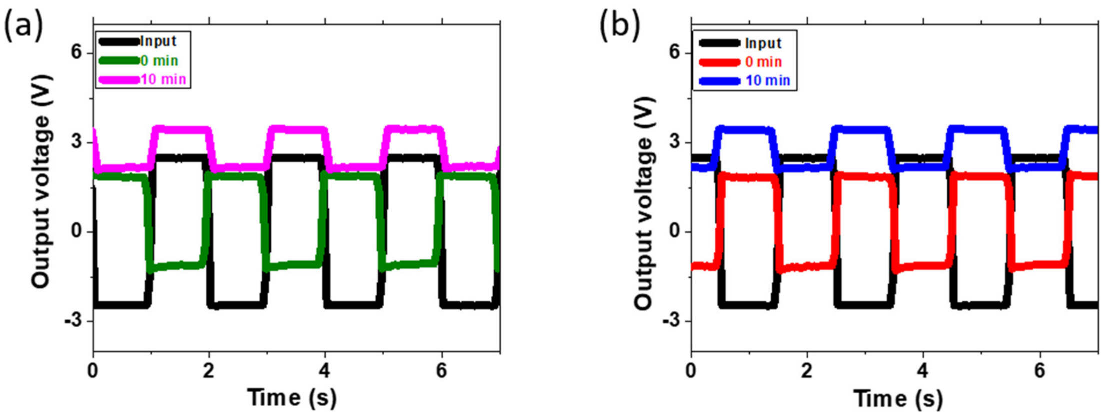

Figure S5. (a) Inverting bare property R2R printed p-type pSWCNT-TFT along with time. (b) Inverting bare property R2R printed n-type pSWCNT-TFT along with time.

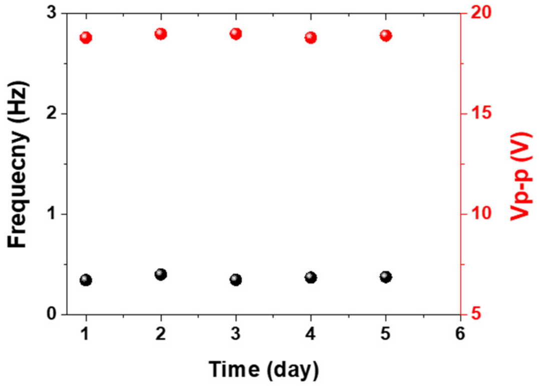

Figure S6. The output frequency with Vp-p from the SiNx passivated 1-bit code generator.

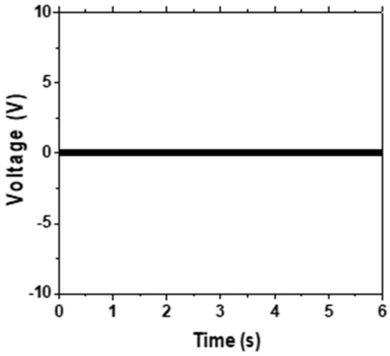

Figure S7. The output characteristics of a non-passivated 1-bit code generator at 85/85 test for 24 hours.
